# Expanding CAR T cells in human platelet lysate renders T cells with in vivo longevity

**DOI:** 10.1186/s40425-019-0804-9

**Published:** 2019-11-28

**Authors:** Alejandro Torres Chavez, Mary Kathryn McKenna, Emanuele Canestrari, Christina T. Dann, Carlos A. Ramos, Premal Lulla, Ann M. Leen, Juan F. Vera, Norihiro Watanabe

**Affiliations:** 1 Center for Cell and Gene Therapy, Baylor College of Medicine, 1102 Bates Avenue, Houston, TX 77030 USA; 2 Cook Regentec, Indianapolis, IN USA

**Keywords:** CAR T cells, Persistence, Memory phenotype, Manufacture, Human platelet lysate

## Abstract

**Background:**

Pre-clinical and clinical studies have shown that the infusion of CAR T cells with a naive-like (T_N_) and central memory (T_CM_) phenotype is associated with prolonged in vivo T cell persistence and superior anti-tumor effects. To optimize the maintenance of such populations during the in vitro preparation process, we explored the impact of T cell exposure to both traditional [fetal bovine serum (FBS), human AB serum (ABS)] and non-traditional [human platelet lysate (HPL) - a xeno-free protein supplement primarily used for the production of clinical grade mesenchymal stromal / stem cells (MSCs)] serum supplements.

**Methods:**

Second generation chimeric antigen receptor with CD28 and CD3ζ endodomain targeting prostate stem cell antigen (PSCA) (P28z) or CD19 (1928z) were constructed and used for this study. After retroviral transduction, CAR T cells were divided into 3 conditions containing either FBS, ABS or HPL and expanded for 7 days. To evaluate the effect of different sera on CAR T cell function, we performed a series of in vitro and in vivo experiments.

**Results:**

HPL-exposed CAR T cells exhibited the less differentiated T cell phenotype and gene signature, which displayed inferior short-term killing abilities (compared to their FBS- or ABS-cultured counterparts) but superior proliferative and anti-tumor effects in long-term in vitro coculture experiments. Importantly, in mouse xenograft model, HPL-exposed CAR T cells outperformed their ABS or FBS counterparts against both subcutaneous tumor (P28z T cells against Capan-1^PSCA^) and systemic tumor (1928z T cells against NALM6). We further observed maintenance of less differentiated T cell phenotype in HPL-exposed 1928z T cells generated from patient’s PBMCs with superior anti-tumor effect in long-term in vitro coculture experiments.

**Conclusions:**

Our study highlights the importance of serum choice in the generation of CAR T cells for clinical use.

## Background

The clinical success of adoptively transferred CD19 targeted chimeric antigen receptor (CAR) modified T cells for the treatment of B cell lymphoma / leukemia has precipitated the extension of this approach to a spectrum of both hematologic malignancies and solid tumors [1, 2]. In parallel, given that in vivo persistence has been shown to correlate with superior outcomes [3, 4], various groups have also explored strategies to enhance T cell longevity ranging from the incorporation of transgenes to support cell expansion (e.g. stimulatory cytokines [5–8] such as IL12 and IL15 or modified cytokine / inhibitory receptor [9–12] to protect cells from the suppressive tumor microenvironment) to manufacturing modifications designed to retain less differentiated T cells (e.g. naïve and central memory T cells) in the infused product. The latter strategy includes the isolation of less differentiated T cell subsets prior to ex vivo activation [13], the incorporation of homeostatic cytokines (e.g. IL7 and IL15 [14]) known to preserve central memory T cells [15–17] for ex vivo expansion, or chemical manipulation of signaling pathways known to be involved in T cell differentiation [18–20], including the activation of Wnt / β-catenin pathway using the GSK3β inhibitor TWS119 [18, 21] or the inhibition of the PI3K/AKT and mTOR pathways using small-molecule inhibitors [22–24]. Though all have proven to effectively enrich for the target T cell populations of interest, the additional complexity (e.g. use of magnetic beads for isolation) and associated costs serve as an impediment to broad clinical implementation.

In the current study we sought to address the issues of complexity and cost by exploring whether T cell differentiation status could be influenced by choice of serum supplementation. Whereas traditional CAR T cell cultures are maintained in fetal bovine (FBS) or human AB serum (ABS)-supplemented medium we investigated the impact of exposure to human platelet lysate (HPL) as an alternative xeno-free protein supplement being used for the expansion of mesenchymal stromal / stem cells (MSCs) in clinic. We now demonstrate in a series of in vitro and in vivo experiments, performed in both hematologic and solid tumor models, the profound qualitative impact of serum supplementation on CAR T cell performance.

## Methods

### Donors and cell lines

Peripheral blood mononuclear cells (PBMCs) were obtained from healthy volunteers and B cell lymphoma and B-ALL patients after informed consent on protocols approved by the Baylor College of Medicine Institutional Review Board (H-15152, H-27471, H-19384 and H-31970). Capan-1 (pancreatic cancer cell line) and DU145 (prostate cancer cell line) were obtained from the American Type Culture Collection (Rockville, MD). NALM6 (pre-B-ALL cell line) and Raji (Burkitt lymphoma cell line) were gifted by Dr. Maksim Mamonkin. Cells were maintained in a humidified atmosphere containing 5% carbon dioxide (CO_2_) at 37 °C. Capan-1 and DU145 cells were maintained in Iscove’s Modified Dulbecco’s Medium (IMDM; Gibco BRL Life Technologies, Inc., Gaithersburg, MD) while NALM6 and Raji cells were maintained in RPMI-1640 media (GE Healthcare Life Sciences, Pittsburgh, PA). Capan-1 cells were grown in IMDM containing 20% heat-inactivated fetal bovine serum (FBS) (Hyclone, Waltham, MA) with 2 mM L-GlutaMAX (Gibco BRL Life Technologies, Inc.) while other cell lines were grown in their specific media containing 10% FBS with 2 mM L-GlutaMAX.

### Generation of retroviral constructs and retrovirus production

A second generation CAR construct targeting PSCA was previously generated in our lab [25]. Briefly, our CAR construct is comprised of scFv domain followed by IgG2 derived-Hinge CH3 spacer with CD28 transmembrane / costimulatory and CD3z signaling domains (P28z). Second generation CAR targeting CD19 was generated based on the P28z construct by replacing the anti-PSCA (clone 2B3) scFv domain with an anti-CD19 scFv (clone FMC63) using restriction enzymes XhoI and BamHI (1928z). The γ-retroviral vectors encoding PSCA-IRES-GFP, GFP/Firefly luciferase fusion protein (GFP/FL) and dominant TGFβ receptor II (DNRII), and the retroviral supernatant were generated as previously described [25–27].

### Generation of CAR-modified T cells and gene-modified cell lines

To generate CAR T cells, 1 × 10^6^ PBMCs were plated in each well of a non-tissue culture-treated 24-well plate pre-coated with OKT3 (1 mg/mL; Ortho Biotech, Inc., Bridgewater, NJ) and anti-CD28 (1 mg/mL; Becton Dickinson & Co., Mountain View, CA) antibodies. Cells were cultured in 10% FBS CTL media [50% RPMI-1640, 50% Clicks medium (Irvine Scientific, Inc., Santa Ana, CA) and 2 mM L-GlutaMAX)], which was supplemented with recombinant human IL2 (50 U/mL, NIH, Bethesda, MD) on day 0. On day 3, retroviral supernatant was plated in a non-tissue culture-treated 24-well plate pre-coated with recombinant fibronectin fragment (FN CH-296; Retronectin; TAKARA BIO INC, Otsu, Japan), and centrifuged at 2000 *g* for 90 min. After removal of the supernatant, OKT3/CD28-activated PBMCs (0.1 × 10^6^/mL) were resuspended in complete medium supplemented with IL2 (100 U/mL) and 2 mL was added to each virus-loaded well, which was subsequently spun at 400 *g* for 5 min, and then transferred to a 37 °C, 5% CO_2_ incubator. On day 3 post transduction, T cells were harvested, washed, and cultured in CTL medium containing different serum supplements - FBS, human ABS (Valley Biomedical, Winchester, Virginia), or pathogen-reduced human platelet lysate (HPL; nLiven PR, Cook Regentec, Indianapolis, IN). In this study, a single lot of HPL was randomly selected as previous work has demonstrated lot-to-lot consistency [28]. Cultures were supplemented with fresh medium and IL2 (50 U/mL) every 2–3 days. To co-express CAR and GFP/FL for in vivo bioluminescence imaging, activated T cells were first modified to express the CAR on day 2 and transduced with GFP/FL on day 3 using the same protocol. Transduction efficiency was measured 3 days post transduction by flow cytometry. To track T cell numbers over time, viable cells were manually counted using trypan blue. To generate tumor cell lines overexpressing PSCA/GFP or GFP/FL, we used the same protocol as previously described and isolated the GFP positive fraction using a cell sorter (SH800S, Sony Biotechnology, San Jose, CA). While T cells were generated in CTL medium containing different serum supplements, all in vitro functional assays were performed in CTL medium supplemented with 10% FBS.

### Genome editing of the CCR7 gene in T cells

Guide RNA for the CCR7 gene (gRNA sequence: GGGCAGGTAGGTATCGGTCA) was designed using CRISPRscan [29] and incorporated into an oligonucleotide primer and used to amplify the gRNA scaffold from PX458 plasmid (gift from Dr. Tim Sauer). gRNA was generated through in vitro transcription with HiScribe™ T7 High Yield RNA Synthesis Kit (New England Biolabs, Beverly, MA) from the DNA template and purified using the RNA Clean & Concentrator kit (Zymo Research, Irvine, CA). Electroporation of 0.25 × 10^6^ T cells was performed in 10 μL of buffer T with 1 μg of gRNA and 1 μg of Cas9 protein (PNA Bio, Newbury Park, CA) by three consecutive 1600 V 10-ms pulses using the Neon Transfection System (Thermo Fisher Scientific, Waltham, MA).

### Flow cytometry

Cells were stained with antibodies for 20 min at 4 °C. All samples were acquired on a Gallios Flow Cytometer (Beckman Coulter Life Sciences, Indianapolis, IN), and data was analyzed using Kaluza 2.1 Flow Analysis Software (Beckman Coulter Life Sciences). Antibodies used in this study are listed in Additional file 1: Table S1.

### RNAseq analysis

Total RNA was extracted from T cells maintained in different serum containing CTL medium using the RNeasy plus Mini kit (QIAGEN, Valencia, CA) and quantified using the NanoDrop 2000 (Thermo Fisher Scientific). RNA sequencing and analysis were performed by Novogene Corporation (Sacramento, CA). Heat map was generated using Heatmapper [30] .

### ^51^Chromium-release assay

The cytotoxicity and specificity of engineered T cells were evaluated in a standard 5 h ^51^Cr-release assay, as described previously [25].

### Degranulation assay

P28z T cells (0.2 × 10^6^ cells) were cocultured with DU145^PSCA^ cells (0.01 × 10^6^ cells, E:T = 20:1) in 200 μL in the presence of Monensin (BD GolgiStop, BD Biosciences, San Jose, CA) and CD107a-APC antibody (H4A3 / 641,581) for 4 h. Similarly, 1928z T cells (0.2 × 10^6^ cells) were cocultured with NALM6 cells (0.2 × 10^6^ cells, E:T = 1:1). Cells were stained for T cell surface markers and the expression of CD107a was analyzed by flow cytometry.

### Cytokine quantification

To measure cytokine production, 0.2 × 10^6^ T cells were cocultured with 0.2 × 10^6^ target cells in 200 μL of medium in a single well of a U-bottom 96-well plate for 24 h. Supernatants were collected and stored at − 80 °C. Cytokine levels were analyzed using MILLIPLEX MAP Human CD8+ T Cell Magnetic Bead Panel Premixed 17 Plex (Merck Millipore, Billerica, MA), according to manufacturer’s instructions.

### Cell proliferation assay and detection of apoptotic cells

T cells were stained with CellTrace Violet (Thermo Fisher Scientific, Invitrogen, Carlsbad, CA) according to the manufacturer’s protocol. The stained P28z T cells and 1928z T cells (0.5 × 10^6^ cells) were cocultured with either Capan-1^PSCA^ cells (0.1 × 10^6^ cells) or NALM6 (0.5 × 10^6^ cells), respectively, in a 24-well tissue culture plate for 5 days. Cells were harvested and stained for T cell surface markers, Annexin V-APC (BD Bioscience) and 7-AAD (BD Biosciences) and analyzed by flow cytometer.

### Coculture experiments

In the coculture experiments with P28z T cells, 1.25 × 10^4^ Capan-1^PSCA^ cells were plated in 6-well plate on day − 1, then 5 × 10^5^ T cells were added on day 0. For 1928z T cells, 0.1 × 10^6^ 1928z T cells were cocultured with 0.1 × 10^6^ NALM6 cells. Cells were harvested, stained and analyzed by flow cytometer every 3 days. To quantify cells by flow cytometry, 20 μL of CountBright Absolute Counting Beads (Thermo Fisher Scientific, Invitrogen) was added and 7-AAD was added to exclude dead cells. Acquisition was halted at 2000 beads.

### In vivo study

For the subcutaneous (s.c.) tumor model, NOD.Cg-Prkdc^scid^ Il2rg^tm1Wjl^/SzJ mice (NSG mice, Stock No: 005557, 5–7 weeks old, The Jackson Laboratory) were engrafted s.c. (right flank) with Capan-1^PSCA^ cells (5 × 10^6^ cells / animal) and once the tumors were established (day 21) the animals were treated with 1 or 2 × 10^6^ of P28z T cells engineered to express GFP/FL intravenously. For tumor rechallenge, 5 × 10^6^ Capan-1^PSCA^ cells were injected on left flank on day 42 post T cell administration. Tumor size was measured by calipers and tumor volume was calculated as follows: tumor volume (mm^3^) = length x width^2^ / 2. T cell migration and distribution were evaluated by injecting mice intraperitoneally with 100 μL of D-luciferin (15 mg/mL, PerkinElmer Inc., Waltham, MA) followed by bioluminescence imaging using an IVIS Lumina II imaging system (Caliper Life Sciences, Inc., Hopkinton, MA), and analyzed by Living Image software (Caliper Life Sciences, Inc.). To assess PSCA expression on residual tumor, mice were sacrificed, tumors were dissected, and single cell suspensions were prepared, as previously published [25]. Subsequently, cells were stained with either anti-PSCA or isotype control followed by Rat anti-mouse IgG1-APC. To distinguish Capan-1 cells, cells were further stained with anti-EpCAM-PE antibody and 7-AAD to exclude dead cells. For the systemic tumor model, 0.5 × 10^6^ NALM6 cells engineered to express GFP/FL were injected into NSG mice intravenously on day − 3, then 5 or 10 × 10^6^ 1928z T cells were injected intravenously. To quantify T cells in the mouse peripheral blood, 50 μL of blood obtained by submandibular facial vein bleeding was stained with CD3, CD4, CD8, and CD45, then treated with RBC Lysis Buffer (BioLegend, San Diego, CA) to lyse red blood cells. CD45^+^CD3^+^ cells were counted by flow cytometer using CountBright Absolute Counting Beads. In the experiment to track T cell migration and expansion, mice were injected with 0.5 × 10^6^ NALM6 cells followed by 5 × 10^6^ 1928z T cells modified to express GFP/FL. All in vivo experiments were performed according to the Baylor College of Medicine Animal Husbandry guidelines with approval from the Institutional Animal Care and Use Committee (IACUC).

### Statistical analysis

Statistical analysis was performed using Graphpad Prism 7 software (GraphPad Software, Inc., La Jolla, CA). The statistical analysis used in each experiment is described in the figure legend.

## Results

### Expanding CAR T cells in HPL results in maintenance of a less differentiated T cell phenotype

**Fig. 1:**
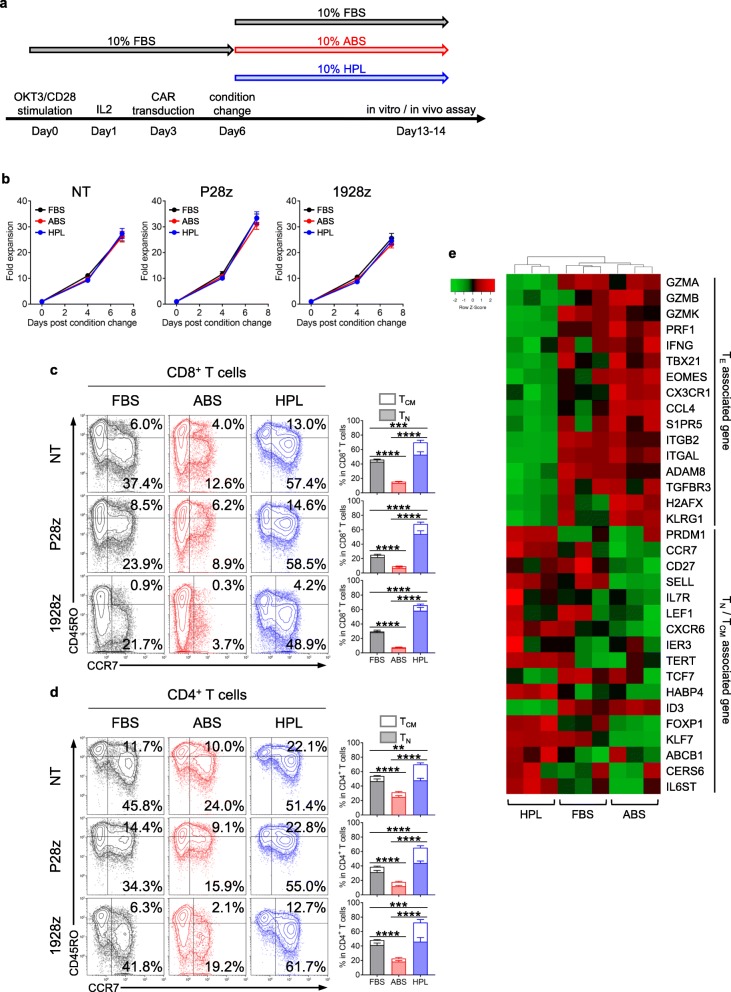
Characteristics of T cells expanded in different serum component. (**a**) Schema of CAR T cell generation. (**b**) T cell expansion after changing serum supplement (mean ± S.E., *n* = 14 for NT, *n* = 12 for P28z, *n* = 9 for 1928z). (**c**, **d**) T cell phenotype after 7 days expansion in different serum. Representative dot plots show CCR7 and CD45RO expression in CD8^+^ T cells (**c**) and CD4^+^ T cells (**d**). Bar graph summarizes the result of multiple donors. Each empty and filled square indicate T_CM_ and T_N_ cells, respectively (mean ± S.E., n = 9 for NT, n = 12 for P28z, *n* = 7 for 1928z). (**e**) Heatmap showing T_CM_ / T_N_ and T_E_ associated gene expression in P28z T cells expanded in different serum component from 3 donors. Statistical differences are calculated by Two-way ANOVA with Tukey multiple comparison (**b**) or One-way ANOVA with Tukey multiple comparison (c, d). *p ≤ 0.05, **p ≤ 0.01, ***p ≤ 0.001, ****p ≤ 0.0001

To evaluate the impact of different sera on CAR T cell function during the cell expansion phase, we first transduced OKT3/CD28 stimulated T cells cultured in medium supplemented with 10% FBS with a retroviral vector encoding either a second generation prostate stem cell antigen (PSCA)-targeted CAR or a CD19-specific CAR, each containing the endodomains CD28 and CD3z (P28z and 1928z, respectively). Three days after transduction (transduction efficiency: P28z - 87.8 ± 1.5%; *n* = 14, 1928z - 90.9 ± 1.5%; *n* = 9. Additional file 2: Figure S1a), the T cell cultures were split into three conditions and cultured in medium supplemented with either 10% FBS, 10% ABS or 10% HPL (Fig. 1a) and further expanded for an additional week in the presence of IL2. We first evaluated the impact of differential sera exposure on T cell expansion and found that there was no statistical difference between the 3 conditions (Fig. 1b). However, when we examined the phenotypic profile of the expanded cells we found that those cultured in HPL showed a trend towards increased CD4^+^ T cell numbers (Additional file 2: Figure S1b) as well as a significantly higher percentage of CCR7^+^ cells representing naïve-like (T_N_) and central memory T cells (T_CM_) in both CD8^+^ and CD4^+^ T cell fractions, independent of CAR construct (Fig. 1c and d). We also explored the expression of other cell surface makers associated with memory (CD62L, CD127, CD27 and CD28), activation (CD25 and CD69) and inhibition (PD1 and TIM3). HPL-expanded cells showed increased expression of CD25, CD69, PD1 and TIM3 (Additional file 2: Figure S1d), whereas cells expanded in ABS showed lower expression of CD62L^+^ in both CD8^+^ and CD4^+^ T cell subsets as well as diminished numbers of CD27^+^CD28^+^ populations in CD4^+^ T cells across non-transduced T cells (NT), P28z and 1928z (Additional file 2: Figure S1c). Of note, we discovered that cultures could tolerate lower HPL concentrations (as low as 2.5%) without disruption of T cell growth or phenotype, unlike FBS where reduction to below 5% impeded T cell expansion (Additional file 2: Figure S2). We extended our characterization of P28z-modified cells by performing RNAseq analysis (Day 7 post-media change) where we found that cells expanded in HPL highly expressed T_N_ / T_CM_-related genes such as LEF1, FOXP1 and KLF1 and less effector T cell (T_E_)-related genes encoding transcription factors such as TBX21, EOMES and KLRG1, as well as effector molecules such as granzyme, perforin and IFNγ [31–33] (Fig. 1e). Thus, cultures expanded in HPL appeared to be enriched in cells that exhibit T_N_/T_CM_ characteristics based both on phenotypic and gene expression profiling studies.

### Effector function of CAR T cells expanded in HPL

**Fig. 2:**
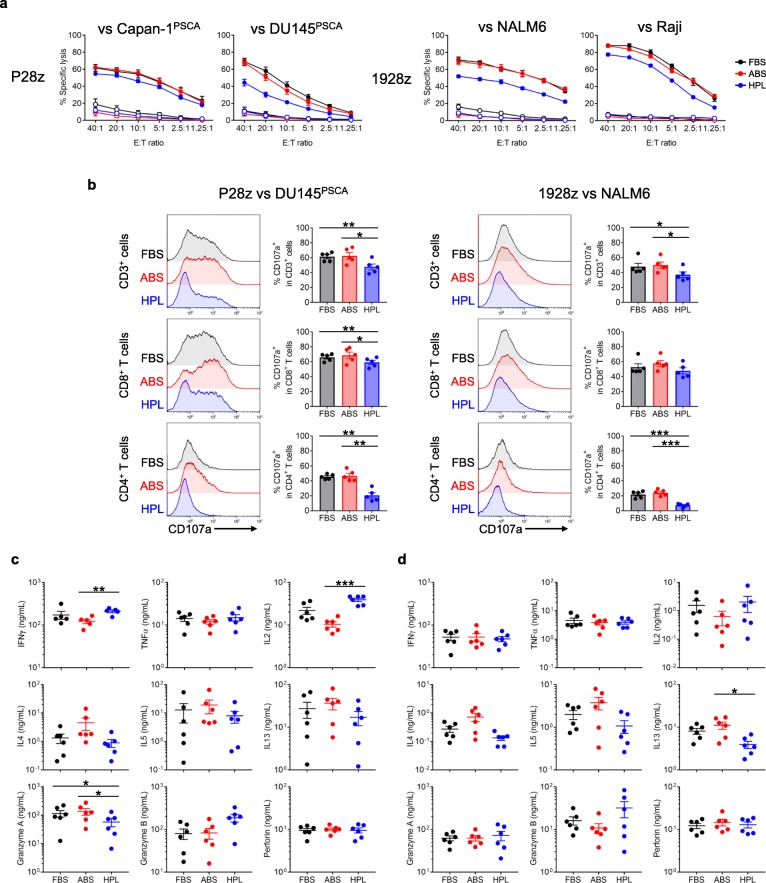
Effect of serum on cytotoxicity and cytokine production of CAR T cells. (**a**) ^51^Cr-release assay showing cytotoxicity of P28z T cells against Capan-1^PSCA^ (*n* = 3) and DU145^PSCA^ (n = 7), and 1928 T cells against NALM6 (*n* = 4) and Raji (n = 3) (mean ± S.E.). (**b**) CD107a degranulation assay. P28z or 1928z T cells are cocultured with DU145^PSCA^ or NALM6, respectively, for 4 h with CD107a staining. Each representative histogram shows CD107a expression on either CD3^+^, CD8^+^ or CD4^+^ cells. Bar graph summarize the result from 5 donors (mean ± S.E). (**c**, **d**) Cytokine production from CAR T cells. P28z (**c**) or 1928z (**d**) T cells are cocultured with either Capan-1^PSCA^ or NALM6, respectively, for 24 h. Cytokines secreted in supernatant are measured by Multiplex (mean ± S.E., *n* = 6). Statistical differences are calculated by Two-way ANOVA with Tukey multiple comparison (**a**) or One-way ANOVA with Tukey multiple comparison (**b**, **c**, **d**). *p ≤ 0.05, **p ≤ 0.01, ***p ≤ 0.001, ****p ≤ 0.0001

We next evaluated the function of CAR T cells exposed to different sera conditions. First to assess short-term cytotoxicity, we performed a 5 h ^51^Cr-release assay by coculturing P28z T cells with Capan-1 and DU145 cell lines modified to overexpress PSCA, and coculturing 1928z T cells with NALM6 and Raji cell lines. As shown in Fig. 2a, CAR T cells expanded in HPL showed slightly but significantly lower killing ability compared to FBS- and ABS-supplemented cultures. Since HPL cultures contain higher frequencies of less-differentiated T cells, we also evaluated degranulation of CAR T cells in select culture conditions, P28z vs DU145^PSCA^ and 1928z vs NALM6. Upon antigen engagement HPL-expanded cells exhibited lower frequencies of CD107a^+^ cells (P28z: FBS - 61.3 ± 2.6%, ABS - 62.5 ± 4.6%, HPL - 47.7 ± 3.9%, 1928z: FBS - 47.8 ± 4.6%, ABS - 50.1 ± 4.1%, HPL - 37.2 ± 3.8%, mean ± S.E., *n* = 5) - a phenomenon detected in both the CD8^+^ and most notably in the CD4^+^ T cell fraction (Fig. 2b). We further investigated CD107a expression by sub-fractionating the CD8^+^ T cell compartment based on CCR7 expression. As shown in Additional file 2: Figure S3a, we found that the frequency of CD107a^+^ cells was similar within the CCR7^−^ or CCR7^+^ fractions of the different culture conditions, but the CCR7^−^ fraction showed a higher degree of degranulation than the CCR7^+^ fraction. Thus, the lower short-term cytotoxicity observed in the HPL cultures could be ascribed to: i) significantly less degranulation of CD4^+^ T cells in both CCR7^−^ and CCR7^+^ fraction (Additional file 2: Figure S3b), and ii) enrichment of CCR7^+^ cells within the CD8^+^ T cell compartment within HPL cultured populations, which inherently show less degranulation than the CCR7^−^ fraction. We next evaluated cytokine production. Twenty-four hours post CAR stimulation HPL-cultured P28z T cells produced higher levels of IFNγ and IL2 compared to ABS cultures, though this trend was not observed with 1928z T cells. Additionally, there was a trend towards diminished production of the Th2 cytokines IL4, IL5 and IL13, as well as increased Granzyme B in the HPL-supplemented cultures (Fig. 2c and d).

### HPL cultured CAR T cells showed a higher proliferative capacity leading to potent anti-tumor response in long-term in vitro coculture experiments

**Fig. 3:**
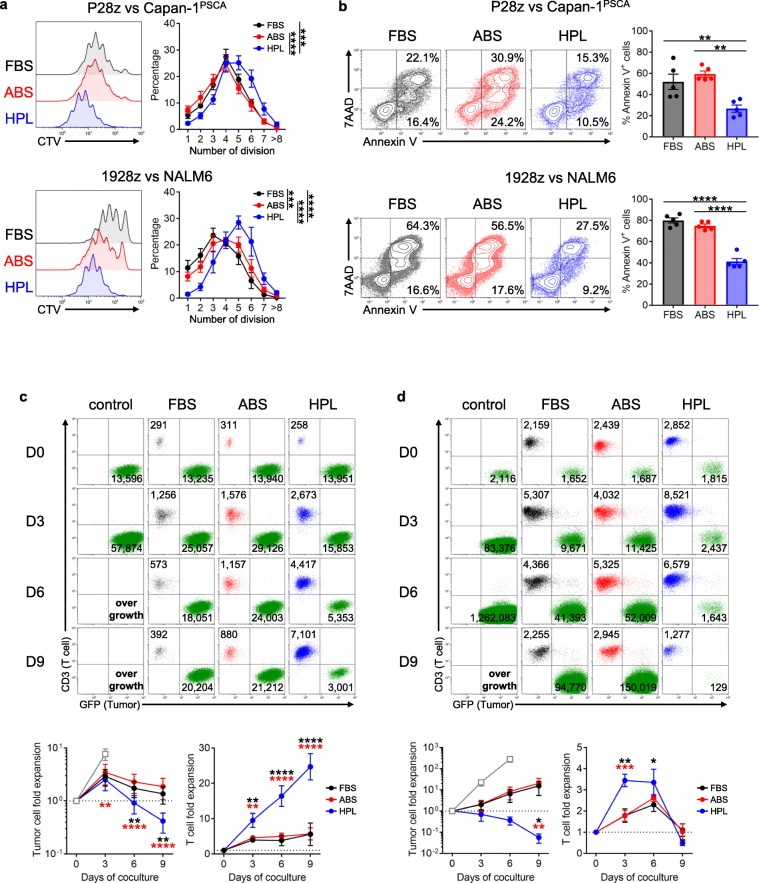
Effect of serum on proliferation and anti-tumor response of CAR T cells. (**a**, **b**) Cell proliferation assay using CellTrace Violet (CTV) and detection of apoptotic cells. P28z or 1928z T cells were stained with CTV and cocultured with either Capan-1^PSCA^ or NALM6, respectively, for 5 days. After coculture, T cells were stained with Annexin V and 7AAD. Dilution of CTV (**a**) and apoptosis (**b**) were analyzed by flow cytometry. Histogram and dot plot show representative data and graph summarize the result from 5 donors (mean ± S.E.). (c, d) In vitro long-term coculture experiment. P28z T cells (1.25 × 10^4^ cells) were cocultured with pre-plated 5 × 10^5^ Capan-1^PSCA^ cells (**c**) or 1928z T cells (1 × 10^5^ cells) were cocultured with 1 × 10^5^ NALM6 cells (**d**) for 9 days. Cells were collected every 3 days and counted by using counting beads on flow cytometry. Number in representative dot plot indicate cell number of T cells or target cells with 2000 bead count. Graph summarizes the result from 6 donors (mean ± S.E.). Statistical differences are calculated by Two-way ANOVA with Tukey multiple comparison (a, c, d) or One-way ANOVA with Tukey multiple comparison (**b**). *p ≤ 0.05, **p ≤ 0.01, ***p ≤ 0.001, ****p ≤ 0.0001

Since T_N_ and T_CM_ populations (which were enriched in the HPL condition - Fig. 1c and d) have been shown to possess higher proliferative capacity upon antigen stimulation compared to their T_E_ counterparts [13, 31], we hypothesized that HPL-exposed CAR T cells would exhibit superior proliferative capacity compared with the other serum conditions. Thus, we labeled either P28z or 1928z T cells from each of the serum conditions with CellTrace Violet dye (CTV) and subsequently stimulated them with Capan-1^PSCA^ or NALM6 cells, respectively. After 5 days in culture, we evaluated cell proliferation based both on CTV dilution as well as CAR T cell apoptosis (Annexin V and 7AAD staining). As shown in Fig. 3a, HPL-expanded P28z and 1928z T cells showed a significantly higher number of cell divisions upon antigen stimulation and less apoptosis (P28z - Annexin V^+^ apoptotic cells: FBS - 52.1 ± 7.2%, ABS - 59.4 ± 2.9%, HPL - 26.7 ± 3.4%, mean ± S.E. *n* = 5; 1928z - FBS - 80.0 ± 2.4%, ABS - 74.9 ± 1.7%, HPL - 41.3 ± 2.9%, mean ± S.E., n = 5) (Fig. 3b). Since we observed diminished short-term cytotoxicity (Fig. 2a) but higher proliferative capacity (Fig. 3a) of HPL-expanded CAR T cells, we next evaluated long-term in vitro anti-tumor effects, which rely both on cell proliferation and cytotoxic effects. Thus, we performed a 9-day in vitro coculture experiment in which we simultaneously monitored both tumor cell killing and T cell expansion. At the end of coculture of P28z T cells with Capan-1^PSCA^, HPL-cultured P28z T cells showed potent anti-tumor activity (tumor cell fold expansion: FBS - 1.4 ± 0.5, ABS - 1.9 ± 0.8, HPL - 0.4 ± 0.2, mean ± S.E. *n* = 6) and superior T cell expansion (T cell fold expansion: FBS - 5.6 ± 3.2, ABS - 5.7 ± 1.7, HPL - 24.7 ± 3.7, mean ± S.E. n = 6) (Fig. 3c). Similarly, HPL-cultured 1928z T cells expanded more rapidly and robustly than the other serum conditions (day 3 T cell fold expansion: FBS - 1.8 ± 0.3, ABS - 1.8 ± 0.3, HPL - 3.4 ± 0.3, mean ± S.E. n = 6) leading to the elimination of NALM6 on day 9 (tumor cell fold expansion: FBS - 15.5 ± 10.0, ABS - 20.2 ± 14.7, HPL - 0.06 ± 0.03, mean ± S.E. n = 6) (Fig. 3d). In summary, HPL-expanded CAR T cells in a short term ^51^Cr-release assay exhibit slightly weaker immediate cytotoxic effector function, but when evaluated in a long-term assay their overall tumor killing efficacy is superior to that of CAR T cells expanded in either FBS or ABS due to their higher proliferative capacity.

### HPL-expanded P28z T cells show enhanced in vivo anti-tumor effects

**Fig. 4:**
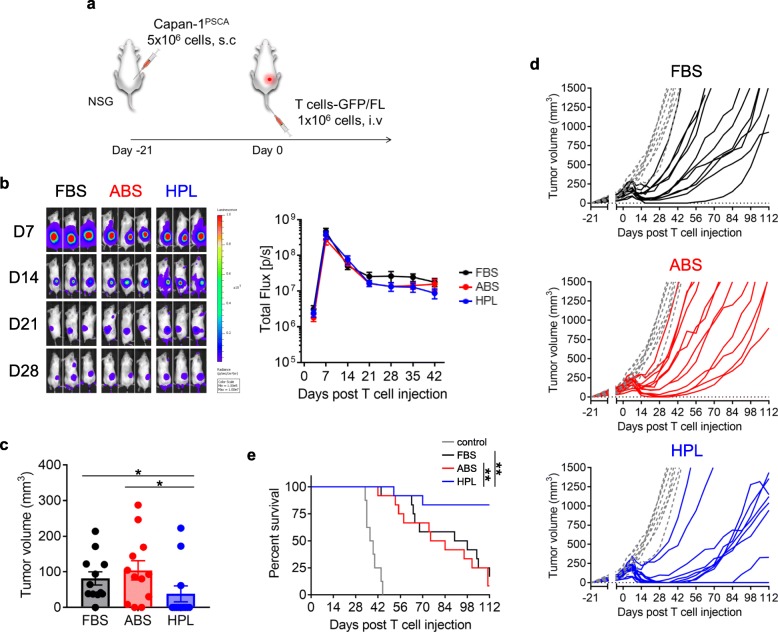
Effect of serum on in vivo performance of P28z T cells. (**a**) Schema of in vivo experiments. (**b**) Representative mice image showing bioluminescence from P28z T cells at different time points. Graph summarizes the results from 12 mice / group (mean ± S.E.). (**c**) Maximum anti-tumor response in each mouse treated with P28z T cell (time range; day 14–28 post T cell infusion). (**d**) Tumor size in individual mice treated with P28z T cells. Gray dotted lines indicate tumor growth with no T cell treatment. (**e**) Survival curve of mice with or without treatment of P28z T cells. Statistical differences are calculated by Two-way ANOVA with Tukey multiple comparison (**b**), an unpaired two-tailed t-test (**c**) or Log-rank test (**e**). *p ≤ 0.05, **p ≤ 0.01

To assess the in vivo anti-tumor effects of CAR T cells cultured in different sera, we engrafted mice s.c. with Capan-1^PSCA^ cells, followed by i.v. administration of GFP/FL-labeled P28z T cells once tumors had reached ~ 100 mm^3^ (approx. 21 days post tumor implantation) (Fig. 4a). Interestingly, we observed similar levels of P28z T cell expansion at the tumor site, irrespective of the culture condition (Fig. 4b). However, when we evaluated the maximum anti-tumor response in each mouse by caliper measurement (ranging from day 10 to day 35 post T cell treatment), HPL-cultured P28z T cell treated animals had superior outcomes (HPL; 38 ± 22 mm^3^ with 9/12 tumor free, FBS; 81 ± 19 mm^3^ with 1/12 tumor free, ABS; 104 ± 27 mm^3^ with 2/12 tumor free, mean ± S.E., *n* = 12) (Fig. 4c). We tracked tumor recurrence up to day 112 post initial CAR T cell treatment and found that 3 mice remained tumor free (Fig. 4d and e), while those that did relapse did so later and was the result of antigen negative relapse (Additional file 2: Figure S4). Given that CCR7^+^ cells were enriched in HPL cultures while expression of other markers conventionally used to discriminate T cell populations (including CD62L, CD127, CD27 and CD28) was not different between the serum groups (Additional file 2: Figure S1c and S1d) this raises the possibility that CCR7 expression is a signature associated with potent anti-tumor activity. However, further investigation of this question disproved the theory since CCR7^KO^ P28z (HPL) T cells exerted similar anti-tumor effects to P28z (HPL) T cells, which was superior to P28z T cells expanded in FBS or ABS (Additional file 2: Figure S5 and Fig. 4d).

**Fig. 5:**
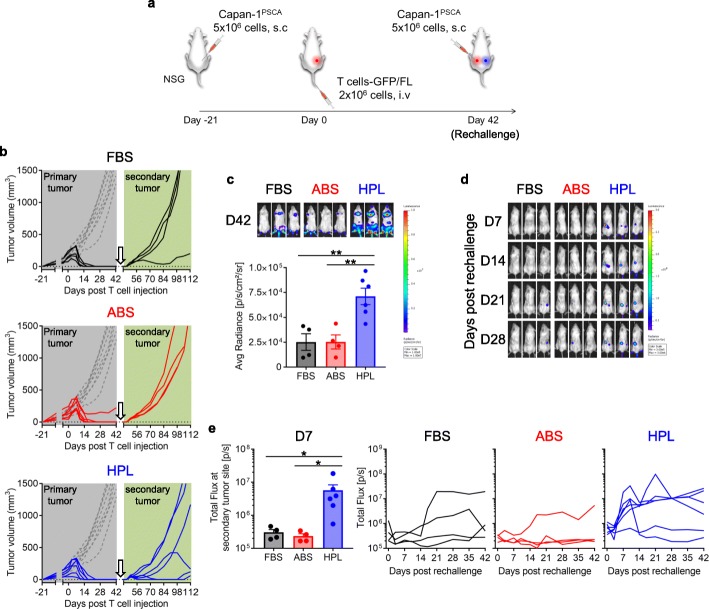
P28z T cell persistence and anti-tumor effect against rechallenged tumor. (**a**) Schema of in vivo tumor rechallenge model. (**b**) Graph indicates tumor volume of primary tumor (gray back ground) and rechallenged tumor (light green background). The arrow indicates the time of tumor rechallenge. (**c**) Representative mice images with T cell bioluminescence on the day of tumor rechallenge (day 42 post T cell injection). Graph summarizes the result of bioluminescence from individual mice (mean ± S.E., n = 4–6). (**d**) Representative mice image showing T cell bioluminescence at different time points. (**e**) Bar graph summarizes T cell bioluminescence at rechallenged tumor site on day 7 post tumor rechallenge from individual mouse and line graph shows T cell bioluminescence at rechallenged tumor site over time (mean ± S.E., n = 4–6). Statistical differences are calculated by One-way ANOVA with Tukey multiple comparison (**c**, **e**). *p ≤ 0.05, **p ≤ 0.01.

To next investigate whether HPL-exposed cells exhibited superior persistence and hence protective capacity in vivo*,* we conducted a tumor re-challenge model whereby mice who cleared their primary tumor [following the administration of a high dose of T cells (2 × 10^6^ T cells / mouse)] were rechallenged with the same tumor cells in the opposite flank (Fig. 5a). Forty-two days after CAR T cell treatment (regardless of serum) we observed that the majority of animals had eliminated their primary tumor (FBS; 5/5, ABS; 5/6, HPL, 6/6) (Fig. 5b, gray background). One of the P28z (FBS) mice was removed from further study due to massive and diffuse in vivo T cell expansion, likely due to xeno-reaction (data not shown). Therefore, 4 P28z (FBS) mice, 5 P28z (ABS) mice and 6 P28z (HPL) mice were re-challenged with tumor cells. One mouse in the P28z (ABS) group had a primary relapse shortly thereafter and was removed from further study. As shown in Fig. 5b (light green background) P28z (HPL) T cell treated mice showed delayed tumor growth (2/6) or complete tumor elimination (2/6) while P28z (FBS) T cell treated mice showed delayed tumor growth only in 1/4 mice while there was no control exerted in the P28z (ABS) mice. We also formally evaluated T cell persistence at the time of tumor rechallenge and found significantly higher bioluminescence from P28z (HPL) T cells (Fig. 5c) indicating longer persistence following primary tumor elimination. Those P28z T cells were able to migrate to the new tumor site (Fig. 5d), expand (Fig. 5e) and exert cytotoxicity resulting in delaying tumor growth or complete tumor elimination.

### HPL-cultured 1928z T cells exhibit higher proliferative capacity resulting in the elimination of NALM6 tumors

**Fig. 6:**
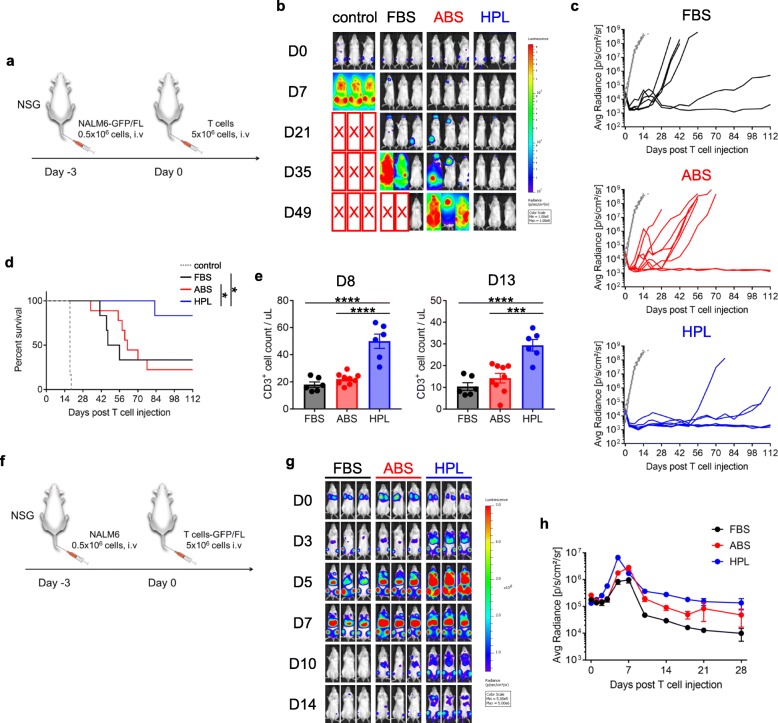
In vivo performance of 1928z T cell expanded in different serum. (**a**) Schema of in vivo experiment for 1928z T cell. (**b**) Representative mice image showing bioluminescence from tumor cells at different time point after 1928z T cell infusion. (**c**) Graph indicate tumor bioluminescence from each mouse treated with 1928z T cells. Gray dotted lines indicate tumor growth with no T cell treatment. (**d**) Survival curve of mice treated with 1928z T cells. (**e**) CD3^+^ cell count in mouse peripheral blood on day 8 and day13 post T cell injection (mean ± S.E., n = 6–9). (**f**) Schema of 1928z T cell in vivo experiment for tracking the migration and expansion of 1928z T cells. (**g**) Mice images show T cell bioluminescence and (**h**) graph summarizes bioluminescence over time (*n* = 3 / group). Statistical differences are calculated by Log-rank test (**d**), One-way ANOVA with Tukey multiple comparison (**e**) or Two-way ANOVA with Tukey multiple comparison (**h**). *p ≤ 0.05, ****p ≤ 0.0001

To investigate whether the superior anti-tumor effects of HPL-CAR T cells extended beyond the Capan-1^PSCA^ model, we conducted a second xenograft mouse model by engrafting mice with GFP/FL+ NALM6 cells (i.v.) followed (3 days later) with 1928z T cells (5 × 10^6^ cells / mouse, i.v.) (Fig. 6a). Regardless of culture condition, all 1928z T cells yielded initial tumor rejection (Fig. 6b, day 7). However, the mice treated with FBS or ABS cultured cells relapsed shortly thereafter while mice treated with HPL-cultured CAR T cells produced prolonged tumor free survival (Fig. 6c and d), which was associated with superior T cell numbers on days 8 and 13 post T cell treatment (Fig. 6e). Since NALM6 tumor cells preferentially localize at the bone marrow and secondary lymphoid organs (Fig. 6b), we also tracked T cell migration and expansion by infusing 1928z T cells transduced with GFP/FL into NALM6-bearing mice (Fig. 6f). As shown in Fig. 6g, 1928z (HPL) T cells rapidly and robustly expanded at sites of disease and persisted longer than 1928z T cells expanded in FBS or ABS (Fig. 6h). These findings were reproduced with the administration of high T cell doses (Additional file 2: Figure S6). Taken together, these data indicate that ex vivo expansion of CAR T cells in HPL enhances in vivo CAR T cell function.

### HPL cultures maintain a less differentiated phenotype of 1928z T cells generated from patients with B cell lymphoma and B cell leukemia

**Fig. 7:**
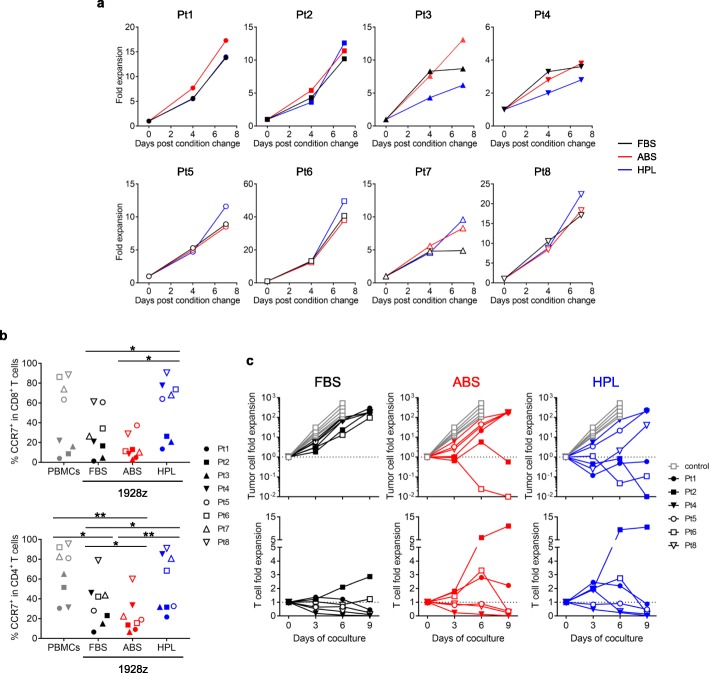
Effect of different sera on characteristics of 1928z T cell generated from patient’s PBMCs. (**a**) 1928z T cell expansion after changing serum component. (**b**) T cell phenotype after 7 days expansion in different serum. Graph summarizes percentage of CCR7^+^ cells in CD8^+^ T cells (left) and CD4^+^ T cells (right). Each symbol indicates each patient samples (*n* = 8). (**c**) In vitro long-term coculture experiment. 1928z T cells (1 × 10^5^ cells/well) were cocultured with 1 × 10^5^ NALM6 cells for 9 days. Cells were collected every 3 days and counted by using counting beads on flow cytometry. Each graph shows fold expansion of tumor cells (top panel) and T cells (bottom panel). Each line indicates each patient samples. Statistical differences are calculated by One-way ANOVA with Tukey multiple comparison (**b**). *p < 0.05, **p < 0.01

Finally, we asked whether HPL could produce similar effects in 1928z T cells generated from patients with B cell lymphoma and B-ALL (Additional file 1: Table S2). We first examined ex vivo expansion and in contrast to healthy donors each patient sample showed different levels of overall expansion (range of fold expansion on day 7 post condition change: FBS; 3.6–40.7, ABS; 3.8–38.0, HPL; 2.8–49.6) (Fig. 7a). Similarly, the memory phenotype was variable. However, HPL-expanded 1928z T cells exhibited a less-differentiated phenotype (CCR7^+^) in both the CD8^+^ and CD4^+^ fractions, similar to the profile seen in healthy donors (Fig. 7b). To evaluate effector function of patient-derived 1928z T cells we performed a long term in vitro coculture experiment with NALM6. As shown in Fig. 7c 4/6 patient samples tested exhibited enhanced anti-tumor responses with higher proliferation of 1928z (HPL) T cells. These results indicate that CAR T cell function is enhanced by simply changing serum supplement from FBS or ABS to HPL.

### TGFβ1 in part plays an important role in maintaining a less differentiated CAR T cell phenotype

In our studies using two different CAR models, HPL-exposed T cells consistently outperformed their ABS or FBS counterparts, leading us to try and identify the component(s) that specifically influenced T cell phenotype. We performed human proteomic analysis of ABS and HPL, and found that HPL contains higher levels of transforming growth factor beta 1 (TGFβ1) compared with ABS (55.4 ± 5.6 vs 2.1 ± 1.1 ng/mL, HPL vs ABS, *n* = 3, in press). Since previous studies have noted that TGFβ1 prevents T cell differentiation and promotes the survival of activated and memory T cells [34–36], to explore the specific effects of TGFβ1 on memory T cell phenotype, we supplemented our FBS and ABS cultures with recombinant TGFβ1 (5 ng/mL) to normalize levels to that seen in 10% HPL cultures. As an additional control we used T cells transgenically expressing a dominant negative TGFβ receptor II (DNRII) [26, 37] to neutralize TGFβ1 in HPL. Interestingly, with TGFβ1 supplementation we observed a higher percentage of CCR7^+^ cells in FBS and ABS cultures, and substitution of DNRII T cells abrogated the effect of HPL on CCR7 expression (Additional file 2: Figure S7a). Taken together, therefore, these results suggest that TGFβ1 has an impact on the maintenance of a less differentiated T cell phenotype. Not surprisingly, though, the killing ability of P28z T cells cultured with recombinant TGFβ1 was impaired in both in vitro and in vivo experiments (Additional file 2: Figure S7b and S7c), suggesting that the combination of various proteins with TGFβ1 contribute to the maintenance of less differentiated T cell phenotype with the retention of effector function.

## Discussion

The correlation between superior clinical outcomes and in vivo T cell persistence has led to the development of various strategies (genetic modification, mechanical isolation, chemical manipulation) designed to preserve/enrich for cells of a less differentiated phenotype within the infusion product. To achieve the same goal with minimal complexity we explored the impact of serum (protein) exposure on CAR T cell phenotype and discovered that simple replacement of traditional FBS or ABS serum with HPL, a GMP-grade xeno-free supplement, arrested CAR T cell differentiation at the T_N_ and T_CM_ phase. HPL-exposed CAR T cells exhibited high proliferative capacity and enhanced long-term in vivo persistence compared to their FBS or ABS counterparts, resulting in superior anti-tumor effects. This data supports the incorporation of HPL in the preparation of clinical grade CAR T cell products for patient administration. Of note, HPL, which is derived from multiple transfusable donors’ platelets, was initially developed to support the ex vivo expansion of MSCs for clinical use in a spectrum of autoimmune diseases including GVHD [38], Crohn’s disease [39], amyotrophic lateral sclerosis [40] and multiple sclerosis [41]. However, to the best of our knowledge, our study is the first to evaluate the effect of HPL-exposure on CAR T cell phenotype or function.

A number of groups have conducted clinical trials using second generation CARs expressing either CD28 or 41BB costimulatory endodomains and correlative studies have demonstrated that the incorporation of CD28 enhances cytotoxicity but is associated with diminished T cell persistence when compared with 41BB [42, 43]. Thus, in the current study we chose to focus on enhancing the longevity of CAR.CD28 T cells using serum supplementation. Using unmodified T cells and T cells modified with two different CAR constructs (P28z and 1928z) we found that cells expanded in HPL contained a significantly higher percentage of less differentiated T cells according to CCR7 expression. Although other makers (e.g. CD62L, CD27 and CD127) frequently used to define memory phenotypes were not different across the serum conditions it should be noted that CCR7 expression (and its associated gene expression profile signature) was not solely responsible for the enhanced anti-tumor effects seen in HPL-exposed cultures given that knocking out this gene did not diminish the effector function of HPL-exposed cells (Additional file 2: Figure S5 - CCR7^KO^ HPL-cultured T cells). Instead, it appears that the less differentiated profile of HPL-exposed P28z T cells, as shown in RNAseq analysis and in vitro proliferation assays, is key in promoting enhanced in vivo anti-tumor effects.

To identify which factor(s) in HPL are responsible for the impact on the T cell differentiation profile we performed proteomic analysis, comparing soluble protein(s) contained in HPL and ABS. However, given the complexity of this serum supplement such assessments have proven challenging. For example, we found that 69 of the 640 proteins assessed were differentially up- or down-regulated by > 10-fold between the two sera [28]. This included TGFβ, which was present in 25-fold greater levels in HPL and importantly has been reported to prevent T cell differentiation [34–36]. In our study we confirmed this finding using recombinant TGFβ1, as shown in Additional file 2: Figure S7a. However, TGFβ1 is immunosuppressive to T cells [44, 45], as highlighted by the detrimental effect of exogenous TGFβ1 on the cytolytic capacity of our CAR T cells (Additional file 2: Figure S7b and S7c), suggesting that the phenotypic and functional characteristics of HPL-exposed CAR T cells is likely a result of multiple soluble proteins, of which TGFβ1 may be one.

## Conclusion

The CAR T cell fields are rapidly growing with the effort to enhance their potency with additional genetic modifications (cytokine, cytokine receptor, switch receptor). The discovery and utilization of gene editing techniques such as ZFN, TALEN and CRISPR/Cas9 should further accelerate the development of new generation of CAR T cells (knock out inhibitory receptor [46–48], knock in CAR into specific locus [49, 50]). Our study highlights the importance of essential culture supplementation in order to improve CAR T cell manufacturing without additional gene modifications. Optimum serum choice can provide improved cellular phenotype for infusion products that may further be improved with the continued advancements in CAR T cell engineering.

## Supplementary information

**Additional file 1: Table S1.** List of antibodies used in this study, **Table S2.** Patient information.

**Additional file 2: Figure S1.** Characteristics of CAR T cells maintained in different sera, **Figure S2.** Effect of lower dose of serum supplement on P28z T cell expansion and phenotype, **Figure S3.** CD107a expression in T cell subsets, **Figure S4.** Tumor status at the time of euthanasia, **Figure S5.** In vivo performance of CCR7KO P28z T cell expanded in HPL, **Figure S6.** In vivo performance of 1928z T cell expanded in different sera, **Figure S7.** Effect of TGFβ1 on T cell phenotype and in vitro / in vivo T cell function.

## Data Availability

The datasets used and/or analyzed during the current study are available from the corresponding author on reasonable request.

## Abbreviations

1928z: Second generation CAR targeting CD19 with CD28 costimulatory domain
ABS: Human AB serum
B-ALL: B lymphoblastic leukemia/lymphoma
CAR: Chimeric antigen receptor
Cas9: CRISPR associated protein 9
CRISPR: Clustered regularly interspaced short palindromic repeats
CTL: Cytotoxic T lymphocyte
CTV: CellTrace Violet dye
DNRII: Dominant TGFβ receptor II
FBS: Fetal bovine serum
FL: Firefly luciferase
GFP: Green fluorescence protein
GMP: Good manufacturing practices
gRNA: guide RNA
GVHD: Graft-versus-host disease
HPL: Human platelet lysate
i.v.: intravenous
IL: Interleukin
IRES: Internal ribosome entry site
MSCs: Mesenchymal stromal / stem cells
NSG: NOD.Cg-Prkdc^scid^ Il2rg^tm1Wjl^/SzJ
NT: Non-transduced T cells
P28z: Second generation CAR targeting PSCA with CD28 costimulatory domain
PBMCs: Peripheral blood mononuclear cells
PSCA: Prostate stem cell antigen
s.c.: Subcutaneous
scFv: Single chain variable fragment
TALEN: Transcription activator-like effector nuclease
T_CM_: Central memory T cells
TE: Effector T cells
TGFβ1: Transforming growth factor beta 1
Th2: Type 2 helper
T_N_: Naïve T cells
ZFN: Zinc-finger nuclease
