# Supplementary material for: Expanding CAR T cells in human platelet lysate renders T cells with in vivo longevity
Source: J Immunother Cancer. 2019 Nov 28;7:330. doi: 10.1186/s40425-019-0804-9 (PMC6883585; doi:10.1186/s40425-019-0804-9)
Supplement: Supplementary file 1 — Additional file 1: Table S1. List of antibodies used in this study, Table S2. Patient information. [file 40425_2019_804_MOESM1_ESM.pdf]

Table S1: List of antibodies used in this study

Name	clone	Product #	Company
CCR7-FITC	150503	561271	BD Biosciences
CD28-PE	CD28.2	561793	BD Biosciences
CD3-PerCP	SK7	347344	BD Biosciences
CD45RO-APC	UCHL1	340438	BD Biosciences
CD25-FITC	2A3	347643	BD Biosciences
PD1-PE	MIH4	557946	BD Biosciences
CD4-APC	SK3	340443	BD Biosciences
CD45-PE	HI30	555483	BD Biosciences
Rat anti-mouse IgG1-APC	X56	550874	BD Biosciences
CD62L-ECD	DREG56	IM2713U	Beckman Coulter Life Sciences
CD27-PC7	1A4CD27	A54823	Beckman Coulter Life Sciences
CD127-APC-AlexaFluor700	R34.34	A71116	Beckman Coulter Life Sciences
CD45RA-APC-AlexaFluor750	2H4LDH11LDB9	A86050	Beckman Coulter Life Sciences
CD8-PacificBlue	B9.11	A82791	Beckman Coulter Life Sciences
CD4-KromeOrange	13B8.2	A96417	Beckman Coulter Life Sciences
CD16-APC-AlexaFluor750	3G8	A66330	Beckman Coulter Life Sciences
CD3-APC-AlexaFluor750	UCHT1	A66329	Beckman Coulter Life Sciences
CD8-PC7	SFCI21Thy2D3	6607102	Beckman Coulter Life Sciences
CD69-ECD	TP1.55.3	6607110	Beckman Coulter Life Sciences
TIM3-APC	F38-2E2	345012	BioLegend
EpCAM-PE	1B7	12-9326-42	Thermo Fisher Scientific, eBioscience
anti-PSCA	7F5	sc-80654	Santa Cruz Biotechnology, Inc.
mouse IgG1	n/a	sc-3877	Santa Cruz Biotechnology, Inc.
Goat anti-human IgG(H+L)-AlexaFluor647	n/a	109-606-088	Jackson ImmunoResearch Laboratories, Inc.

Table S2: Patient information

	sex	age	diagnose	chemotherapy at the drawing blood
Pt1	M	63	adult B cell lymphoma	None
Pt2	M	53	adult B cell lymphoma	None
Pt3	M	65	DLBCL	Fimepinostat
Pt4	M	16	Lymphoblastic lymphoma	None
Pt5	M	17	pediatric B-ALL (Ph negative)	Multidrug chemotherapy (per AALL1331)
Pt6	F	19	pediatric B-ALL (Ph positive)	Ponatinib
Pt7	F	15	pediatric pre-B-ALL	Etoposide / Ifosfamide
Pt8	M	10	pediatric pre-B-ALL	None
