# Supplementary material for: Expanding CAR T cells in human platelet lysate renders T cells with in vivo longevity
Source: J Immunother Cancer. 2019 Nov 28;7:330. doi: 10.1186/s40425-019-0804-9 (PMC6883585; doi:10.1186/s40425-019-0804-9)
Supplement: Supplementary file 2 — Additional file 2: Figure S1. Characteristics of CAR T cells maintained in different sera, Figure S2. Effect of lower dose of serum supplement on P28z T cell expansion and phenotype, Figure S3. CD107a expression in T cell subsets, Figure S4. Tumor status at the time of euthanasia, Figure S5. In vivo performance of CCR7KO P28z T cell expanded in HPL, Figure S6. In vivo performance of 1928z T cell expanded in different sera, Figure S7. Effect of TGFβ1 on T cell phenotype and in vitro / in vivo T cell function. [file 40425_2019_804_MOESM2_ESM.pdf]

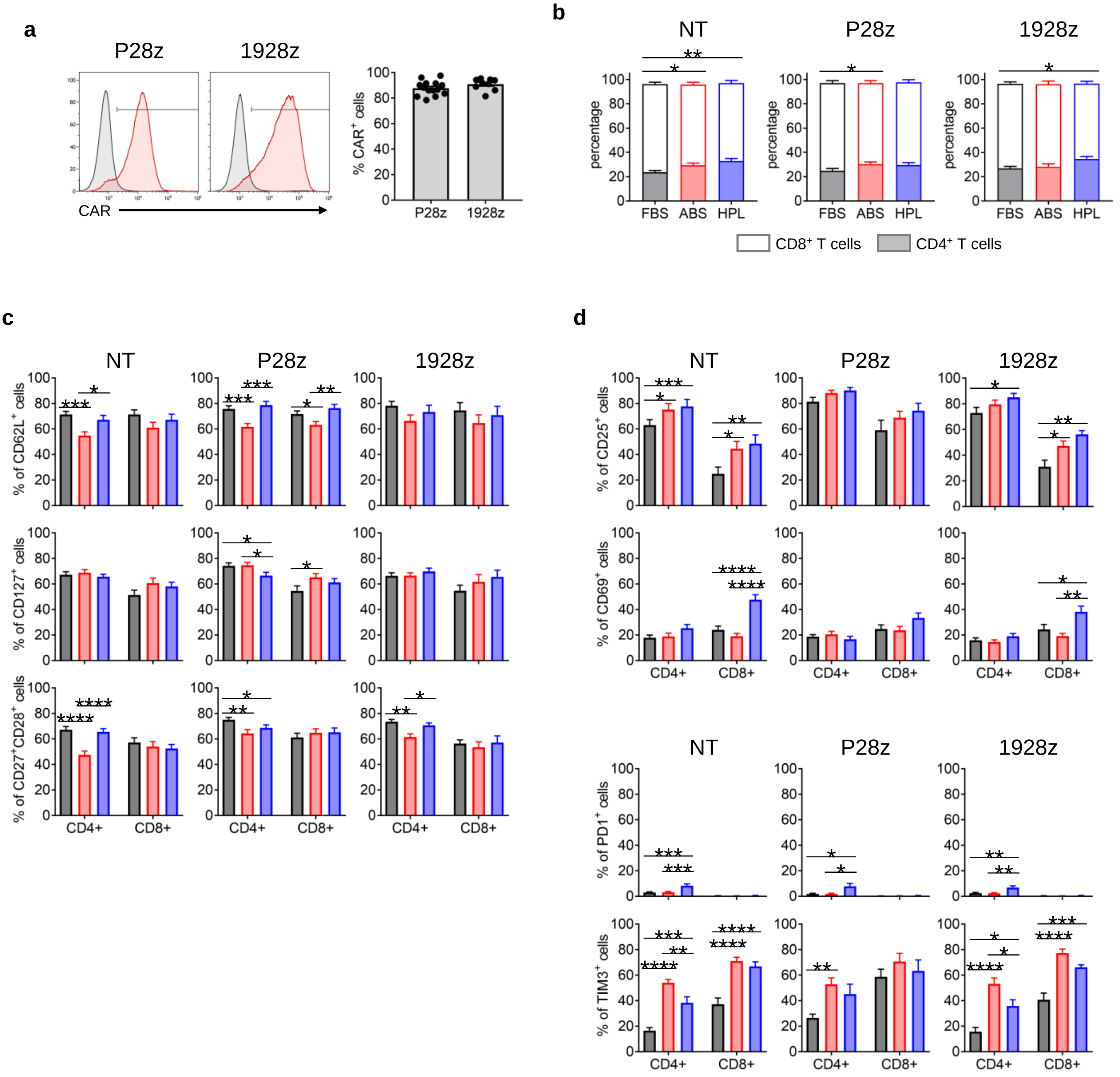

Figure S1: Characteristics of CAR T cells maintained in different sera. (a) Transduction efficiency of P28z and 1928z on day 3 post transduction (n=14 for P28z, n=9 for 1928z). (b) Percentage of CD4 and CD8 positive cells in T cells cultured in different serum supplement (mean ± S.E., n=14 for NT, n=12 for P28z, n=7 for 1928z). (c and d) Surface phenotype analysis of T cell (mean ± S.E., n=14 for NT, n=12 for P28z, n=7 for 1928z). Statistical differences are calculated by One-way ANOVA with Tukey multiple comparison. *p<0.05, **p<0.01, ***p ≤ 0.001, ****p ≤ 0.0001.

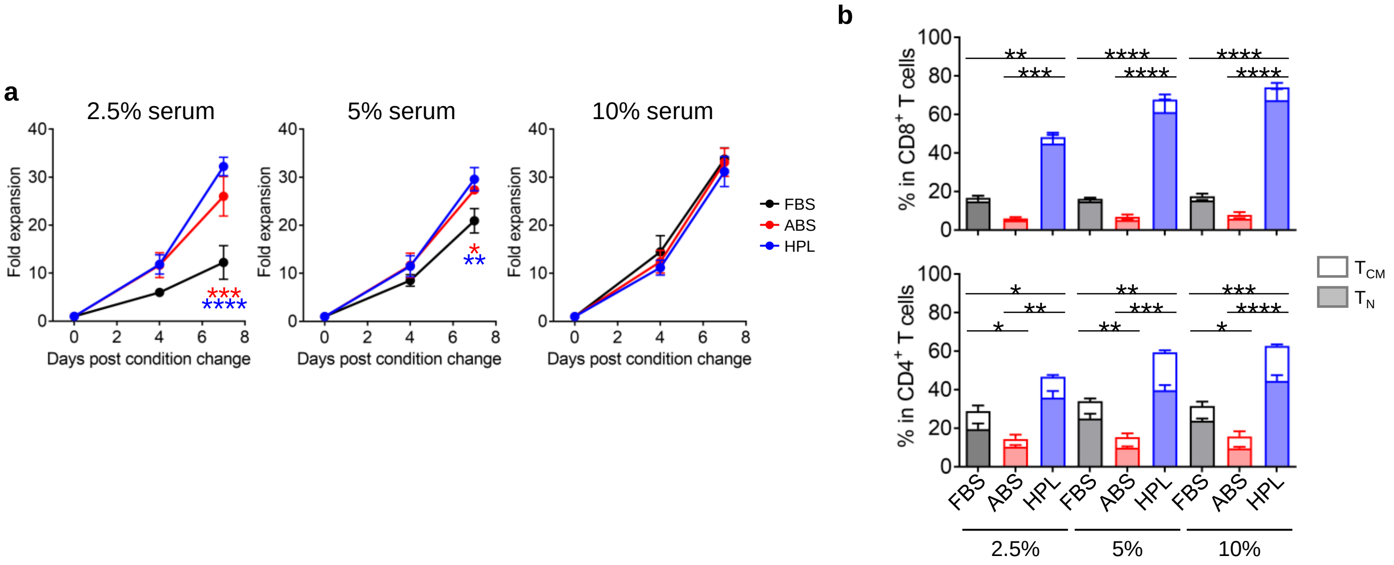

Figure S2: Effect of lower dose of serum supplement on P28z T cell expansion and phenotype. (a) T cell expansion after changing serum supplement at various concentration (mean ± S.E., n=3). (b) T cell phenotype after 7 days expansion in different serum at various concentration (mean ± S.E., n=3). Statistical differences are calculated by Two-way ANOVA (A) or One-way ANOVA (B) with Tukey multiple comparison. *$p \leq 0.05$, **$p \leq 0.01$, ***$p \leq 0.001$, ****$p \leq 0.0001$.

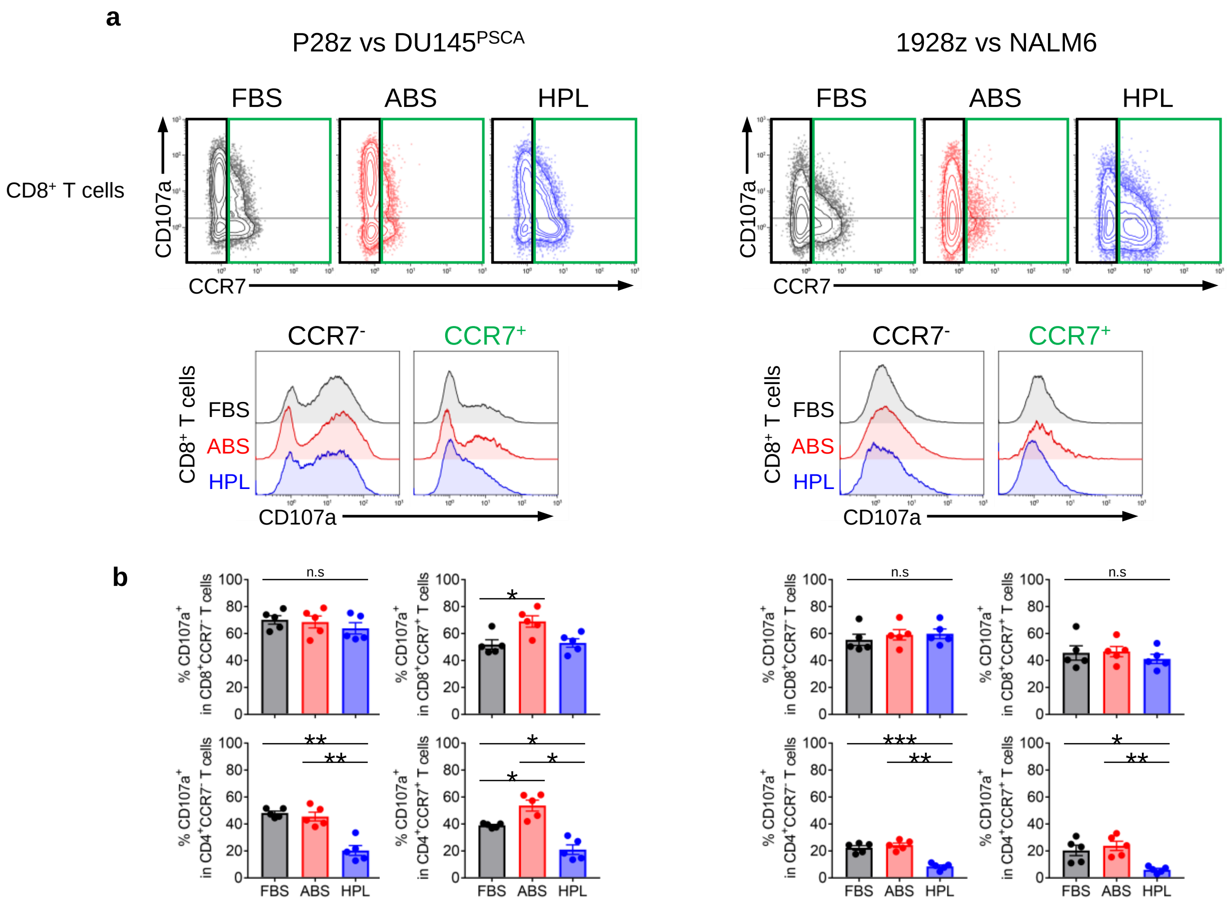

Figure S3: CD107a expression in T cell subsets. (a) Representative dot plot and histogram for CD107a expression in $CD8^+CCR7^-$ and $CD8^+CCR7^+$ fraction. (b) Bar graph summarizes the result of CD107a expression in $CCR7^-$ and $CCR7^+$ fraction in each $CD8^+$ and $CD4^+$ T cells from 5 donors (mean ± S.E). Statistical differences are calculated by One-way ANOVA with Tukey multiple comparison. $^*p<0.05$, $^{**}p<0.01$, $^{***}p \leq 0.001$.

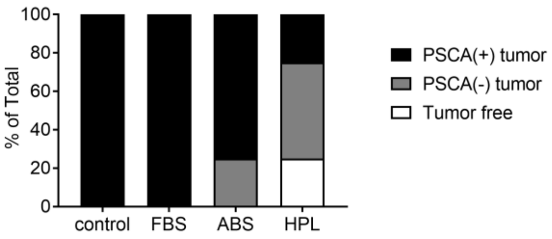

Figure S4: Tumor status at the time of euthanasia.

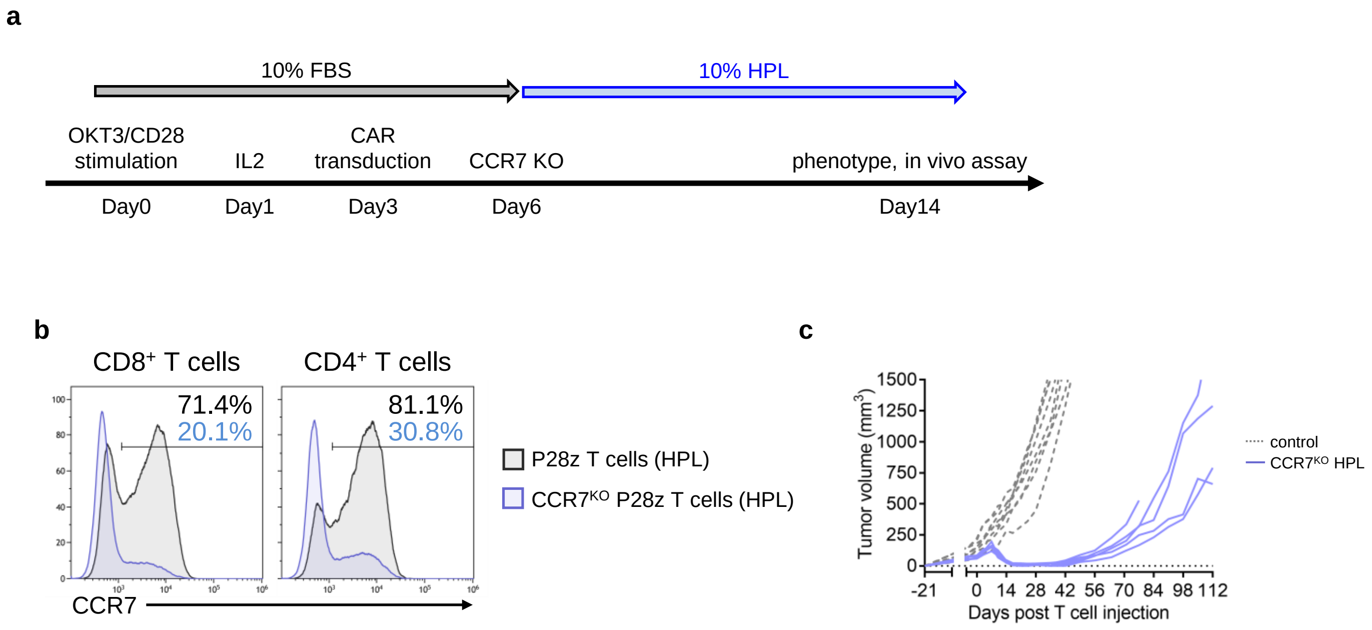

Figure S5: *In vivo* performance of $CCR7^{KO}$ P28z T cell expanded in HPL. (a) Schema of $CCR7^{KO}$ P28z T cell generation. (b) Expression of CCR7 with or without $CCR7^{KO}$. (c) Tumor size in individual mice treated $CCR7^{KO}$ P28z T cells (n=5). Gray dotted lines indicate tumor growth with no T cell treatment shown in Figure 4.

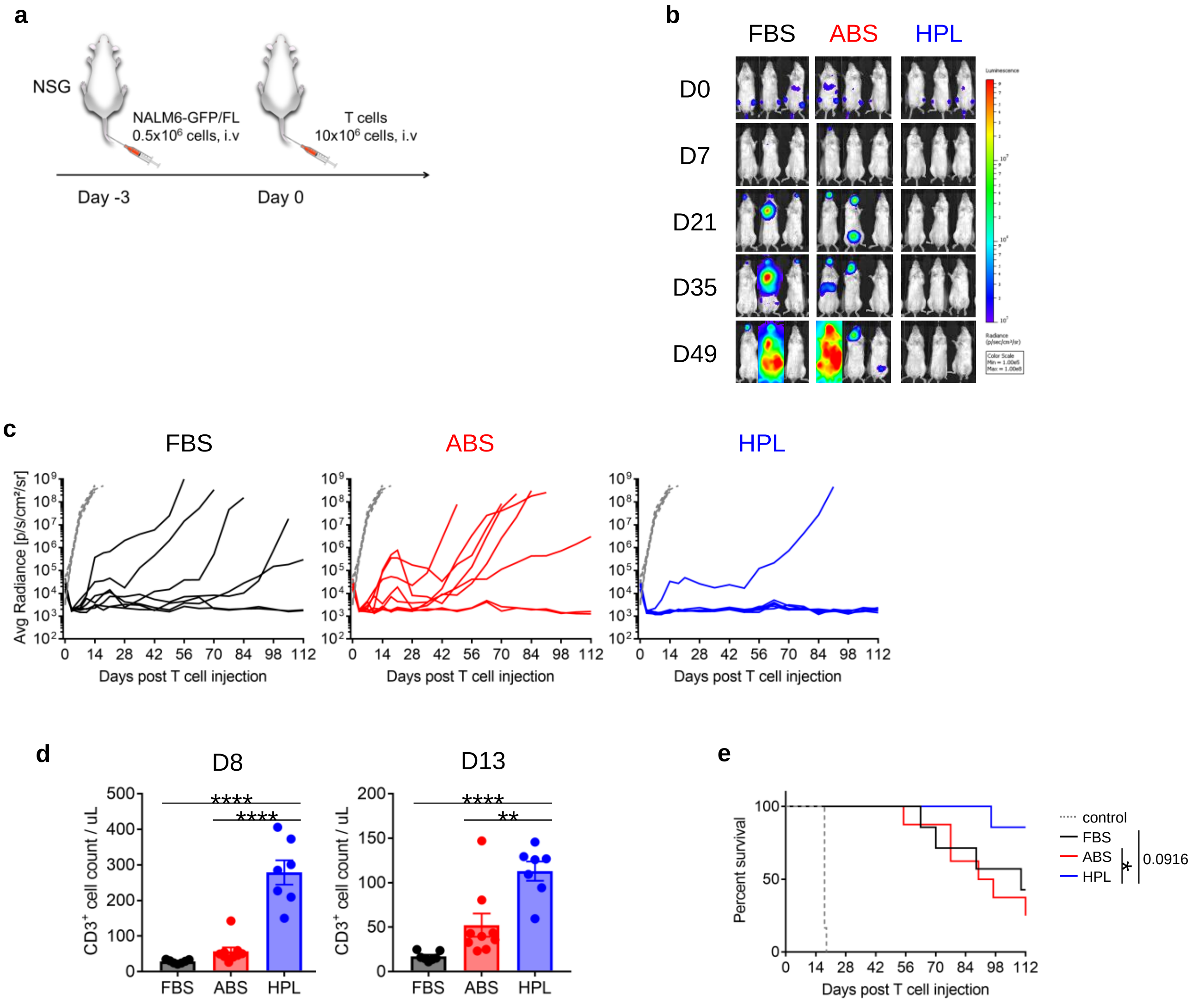

Figure S6: *In vivo* performance of 1928z T cell expanded in different sera. (a) Schema of *in vivo* experiment for 1928z T cell (n=7-8 mice / group). (b) Representative mouse images showing bioluminescence from tumor cells at different time points after 1928z T cell infusion. (c) Graph indicates tumor bioluminescence from each mouse treated with 1928z T cells. Gray dotted lines indicate tumor growth with no T cell treatment. (d) $CD3^+$ cell count in mouse peripheral blood on day 8 and day13 post T cell injection. (e) Survival curve of mice treated with 1928z T cells. Statistical differences are calculated by One-way ANOVA with Tukey multiple comparison (d) or Log-rank test (e). *p<0.05, **p<0.01, ****p ≤ 0.0001.

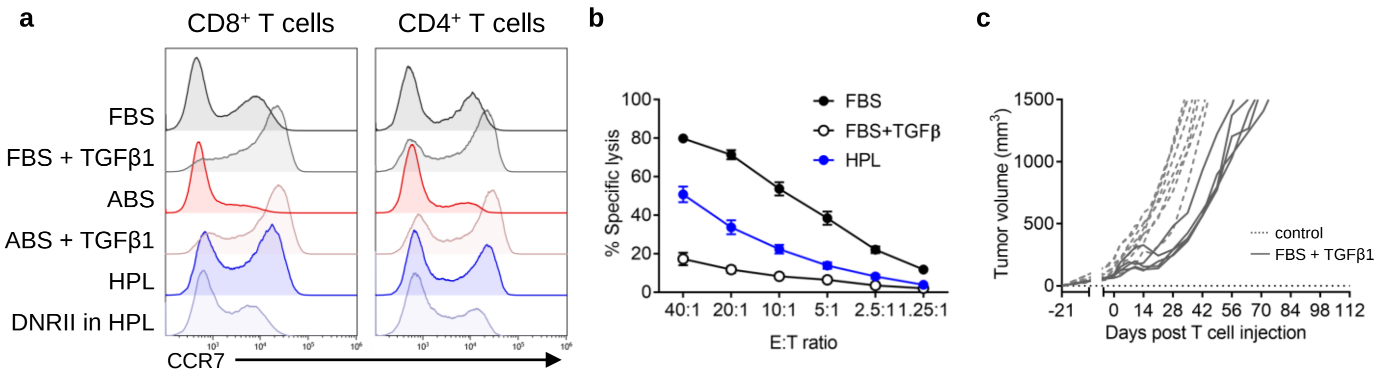

Figure S7: Effect of TGFβ1 on T cell phenotype and *in vitro* / *in vivo* T cell function. (a) CCR7 expression on T cell maintained in different culture conditions. (b) ^{51}Cr-release assay showing cytotoxicity of P28z T cells maintained in different conditions against DU145^{PSCA}. (c) Tumor size in individual mice treated with P28z T cells maintained in 10%FBS + exogenous TGFβ1 (n=5). Gray dotted lines indicate tumor growth with no T cell treatment shown in Figure 4.
